# Complete plastid genome of *Vanda concolor* (Orchidaceae, Aeridinae)

**DOI:** 10.1080/23802359.2019.1679049

**Published:** 2019-10-21

**Authors:** Jin-Liao Chen, Xiong-De Tu, Ding-Kun Liu, Ye Ai

**Affiliations:** Key Laboratory of National Forestry and Grassland Administration for Orchid Conservation and Utilization at College of Landscape Architecture, Fujian Agriculture and Forestry University, Fuzhou, China

**Keywords:** Vanda, plastid genome, phylogeny

## Abstract

*Vanda* is one of the five most horticulturally important genera in Orchidaceae. In this study, we assembled the complete plastid genome of an important cultivated species, *V. concolor.* The plastome was 149,474 bp in length, containing a large single-copy region (LSC) of 85,678 bp, a small single-copy region (SSC) of 12,002 bp, and two inverted repeat regions (IR) of 25,897 bp. A total of 127 genes were predicted, including 38 tRNA, 8 rRNA, and 74 protein-coding genes. Phylogenetic analysis of 21 representative plastome of the subtribe Aeridinae suggested *V. concolor* to be sister to *V. brunnea* and that this pair is sister to *Neofinetia*.

*Vanda* R.Br. (Orchidaceae; Epidedroideae; Vandeae), one of the five most horticulturally important genera in Orchidaceae, consists of approximately 74 species collectively distributed from tropical Asia to New Guinea and Australia (Gardiner et al. 2013; Pridgeon et al. 2014). Many species appear to be narrow (island) endemics, with the highest species diversity in the Southeast Asian archipelagos and the Himalayan–Indochinese region (Pridgeon et al. 2014). DNA-based phylogenetic analyses by Gardiner et al. (2013) have provided strong support for the monophyly of *Vanda sensu lato*, incorporating the former genera *Ascocentrum*, *Ascocentropsis*, *Christensonia*, *Eparmatostigma*, and *Neofinetia*. In this study, we first reported the complete plastid genome of the most important cultivated species, *V. concolor.*

Fresh Leaf sample of *V. concolor* was acquired from Shanche Village, Maguan County (23°11′N, 104°27′E), Wenshan Prefecture, Yunnan Province of China, and voucher specimen deposited at The Orchid Conservation and Research Centre of Shenzhen, Guangdong Province of China (specimen code Z.J. Liu 3335). The total genomic DNA was extracted from fresh leaves tissue, with 400 bp randomly interrupted by the Covaris ultrasonic breaker for library construction. The constructed library was sequenced PE150 by Illumina Hiseq 4000 platform, approximately 20GB data generated. Illumina data were filtered by script in the cluster (default parameter: -L 5, -p 0.5, -N 0.1). Paired reads were removed when N content in sequencing reads exceeded 10% of the read base number and the low quality (*Q* ≤ 5) base number in sequencing reads exceeded 50% of the read base number (Yan et al. 2013). Complete plastid genome of *Gastrochilus fuscopunctatus* (GenBank accession KX871233) as reference, the paired-end reads were filtered with GetOrganelle pipe-line (https://github.com/Kinggerm/GetOrganelle) to get plastid-like reads, then the filtered reads were assembled by SPAdes version 3.10 (Bankevich et al. 2012), the final ‘fastg’ were filtered by the script of GetOrganelle to get pure plastid contigs, and the filtered De Brujin graphs were viewed and edited by Bandage (Wick et al. 2015). Then we can get the circle plastomes. Assembled plastid genome annotation based on comparison with *G. fuscopunctatus* by GENEIOUS v11.1.5 (Biomatters Ltd., Auckland, New Zealand) (Kearse et al. 2012). The annotation result was draw with the online tool OGDRAW (http://ogdraw.mpimp-golm.mpg.de/) (Lohse et al., 2013).

The complete plastid genome of *V. concolo*r (GenBank accession MK836105) is 149,474 base pairs (bp) in length, containing a large single-copy (LSC) region of 85,678 bp, a small single copy (SSC) region of 12,002 bp, and two inverted repeat (IR) regions of 25,897 bp. The new sequence possesses total 127 genes, including 74 protein-coding genes, 8 rRNA genes, and 38 tRNA genes. The overall GC content of the chloroplast genome was 36.63%, while the corresponding values of the LSC, SSC, and IR regions are 33.96%, 27.88%, and 43.07%, respectively. To confirm the phylogenetic position of *V. concolo*r, 21 representative species of Aeridinae were aligned using MAFFT v7.307 (Katoh and Standley 2013), and phylogenetic tree constructed by RAxML (Stamatakis 2014) (Figure 1). The ML tree showed that *V. concolor* is sister to *V. brunnea* (GenBank accession MK442937) and that this pair is sister to *Neofinetia* with strong support.

**Figure 1.**
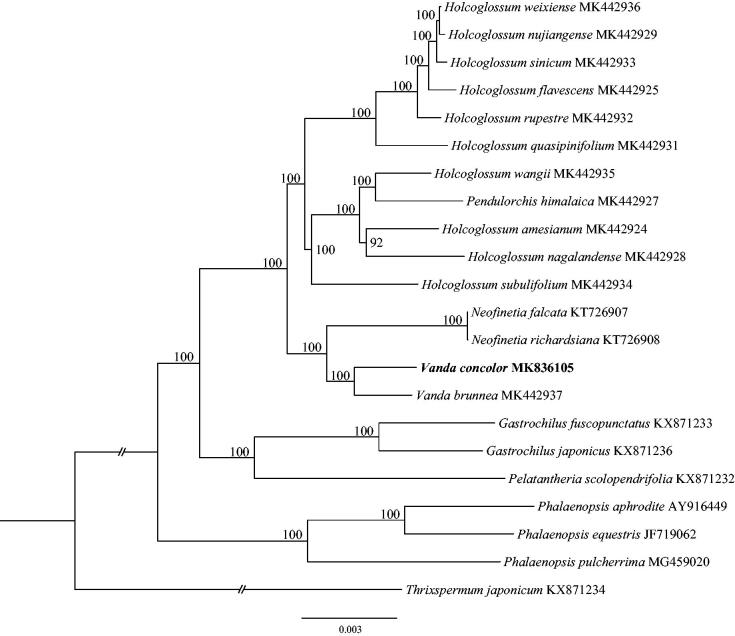
The maximum-likelihood (ML) tree based on the 21 representative plastome genomes of the subtribe Aeridinae. The bootstrap value based on 1000 replicates is shown near each node.
